# Pathways to clinician-scientist identity among medical PhD candidates: a multi-case ideal-typical analysis

**DOI:** 10.1186/s12909-026-09889-y

**Published:** 2026-08-10

**Authors:** Yan Li, Yang Dai, Yongchun Lin

**Affiliations:** 1 College of Education, Zhejiang University, No. 10 Chengjun Yuan, No. 866 Yuhang Tang Road, Xihu District, Hangzhou, Zhejiang 310058 China; 2 School of Political and Public Administration, Wuhan University, No. 299, Bayi Road, Wuchang District, Wuhan City, Hubei 430072 China; 3 School of Public Affairs, #C Building of Information at Zhejiang University, No. 866 Yuhang Tang Road, Xihu District, Hangzhou, Zhejiang 310058 China

**Keywords:** Clinician-scientist identity, Professional identity formation, Medical PhD candidates, Dual-role training, Qualitative multi-case study, Chinese medical education

## Abstract

**Background:**

Clinician-scientists are widely regarded as critical for translational medicine, yet many systems struggle to sustain a stable clinician-scientist workforce. Existing research often assumes a single linear identity pipeline and focuses on Western MD-PhD programs, with limited attention to heterogeneous trajectories in other high-intensity contexts. In China, rapid expansion of dual-role PhD programs in elite “Double First-Class” medical universities has created dense expectations in both clinical service and research. How medical PhD candidates position themselves within these dual demands, and how distinct clinician-scientist identity pathways emerge, remains insufficiently understood.

**Methods:**

A qualitative multi-case study was conducted with 30 medical PhD candidates from 30 top-tier medical universities in North and East China. All participants held a medical degree and were engaged in both clinical and research training. Semi-structured interviews explored motivations, professional self-definition, experiences of dual-role conflict, perceptions of evaluation systems, mentorship arrangements, and access to institutional support. Transcripts were analyzed through inductive coding followed by ideal-typical analysis to construct comparative pathways of clinician-scientist identity development.

**Results:**

Four ideal-typical pathways were identified. The Integrated Dual-Identity type described clinical and research roles as mutually informative and actively sought synergy across settings. The Clinician-First type regarded the PhD primarily as an instrument for clinical career advancement and tended to meet only minimum research requirements. The Research-First type oriented mainly toward a scientific career and substantially reduced clinical engagement during training. The Ambivalent type reported persistent tension between domains, oscillating commitments, and marked psychological strain. Evaluation criteria, mentor configurations, organization of clinical and research time, and availability of institutional resources systematically steered trainees toward particular pathways and rendered some trajectories more fragile than others.

**Conclusions:**

Clinician-scientist identity formation among medical PhD candidates follows multiple pathways. The four ideal types provide an analytic framework for understanding how local structures channel trainees toward integrated dual identities, single-role orientations, or sustained ambivalence. Programs that combine flexible evaluation systems, coordinated clinical and research mentorship, structured protection of time for both roles, and targeted support for ambivalent trainees may strengthen the clinician-scientist pipeline in China and offer transferable design principles for similar high-pressure training environments worldwide.

**Supplementary Information:**

The online version contains supplementary material available at https://doi.org/10.1186/s12909-026-09889-y.

## Introduction

Clinician-scientists, physicians who engage in both patient care and research, are widely regarded as essential for bridging the gap between laboratory discoveries and clinical practice [1, 2]. Around the world, academic medical centers have established dual training programs (such as MD-PhD and similar pathways) to cultivate clinician-scientists capable of driving translational research and innovation in healthcare [3]. However, despite these efforts, many countries face a decline in the number of new clinician-scientists entering the workforce [4]. Long training durations, intense competition for research funding, and the challenge of balancing two demanding career roles have contributed to what some have called a global “pipeline problem” for clinician-scientists [5, 6]. In this context, understanding how emerging clinician-scientists form their professional identity (combining the roles of healer and investigator) has become an important area of inquiry [7, 8].

Developing a professional identity as a clinician-scientist is inherently complex [9]. Medical trainees must master clinical competencies while cultivating scientific reasoning and research skills, often during the same period of training [10, 11]. Early-career clinician-scientists can experience tension between these dual identities [12]. They may struggle with feeling “in-between” the clinical and academic worlds, sometimes lacking a full sense of belonging in either [13]. For example, physicians pursuing a PhD might feel their clinical colleagues view them as too research-oriented, while in the laboratory they may feel like outsiders due to their clinical background [14]. These identity tensions can manifest as imposter syndrome, role overload, or uncertainty about career direction [15, 16]. A well-integrated clinician-scientist identity, in which physician and scientist roles are understood as complementary, is thought to be crucial for sustaining a successful career that advances patient-oriented research [17–19].

China provides a compelling context to study clinician-scientist identity formation [20, 21]. In recent years, Chinese policymakers and top universities have placed new emphasis on training clinician-scientists as part of building world-class medical education and research [22]. China’s “Double First-Class” initiative, for instance, has designated a group of elite universities (including leading medical schools in North and East China) to develop top-tier programs and research output [23, 24]. Many of these institutions have launched integrated medical training programs (such as 8-year MD-PhD tracks and other combined degree initiatives) to produce graduates proficient in both clinical medicine and scientific research [25]. Despite these efforts, the development of clinician-scientists in China remains nascent and faces significant structural challenges. Traditionally, hospital career advancement in China has prioritized clinical service and patient volume, leaving limited room for research activities [26, 27]. Early-career doctors pursuing PhDs often encounter heavy clinical workloads, strict publication requirements for graduation or promotion, and a scarcity of role models who successfully bridge the two domains [28, 29]. These conditions make it difficult for medical PhD trainees to reconcile their dual roles [30]. Thus, examining how Chinese medical PhD candidates navigate and construct their professional identities can shed light on broader issues of clinician-scientist development under such high-pressure, dual-expectation environments [30, 31].

This study aims to explore the pathways through which medical PhD candidates in China’s top-tier universities develop a clinician-scientist identity. We focus on PhD trainees who already hold medical qualifications and are undergoing research training at elite “Double First-Class” medical universities in North and East China. Using qualitative interviews and an ideal-typical analysis, we seek to identify distinct patterns (ideal types) of identity formation among these future clinician-scientists. By constructing and comparing these ideal types, we intend to illuminate how different combinations of personal motivation, mentorship, institutional pressures, and role experiences can lead to divergent identity development trajectories. Ultimately, this work can inform strategies to better support clinician-scientist trainees in China and internationally by addressing the specific needs and challenges associated with each trajectory.

## Methods

### Study design and sample

This research was designed as a qualitative multi-case study of medical PhD candidates who were concurrently positioned within clinical and research training [32]. The institutional context was China’s Double First-Class initiative (Shuang Yiliu), a national designation introduced in 2017 and revised through periodic review. The second-round national list includes 147 universities and identifies selected world-class universities and world-class disciplines; the list does not constitute a closed roster of medical schools [33, 34]. Within this national policy field, our sampling frame comprised Double First-Class universities in North and East China that offered doctoral training in clinical medicine or closely related medical fields and had institutional arrangements linking doctoral study with clinical training. Thirty institutions in these two regions met these criteria and were included in the study; the number therefore refers to the regional sampling frame used in this research, not to the total number of Double First-Class medical institutions nationally. North and East China were selected because these regions contain dense concentrations of tertiary hospitals, biomedical laboratories, and national research universities. The Double First-Class designation was analytically important because the designation intensified expectations for research productivity while clinical departments continued to evaluate trainees through service, rotation, and specialty training requirements. For doctoral trainees located in such settings, clinical evaluation rules and research evaluation rules converged at the same career stage.

A multi-case design was adopted because ideal-type construction requires comparison across cases that vary in institutional context, mentor configuration, evaluation pressure, and clinical specialty. We sampled one participant per institution to prioritize cross-institutional variation over within-site saturation. The design allowed each institution to function as a bounded case environment while each participant’s narrative served as the empirical unit for analyzing clinician-scientist identity formation. Additional participants within a smaller number of institutions would have deepened local description, but the present research question required broader organizational contrast. Restricting the study to high-intensity Double First-Class settings strengthened the analytic comparison because identity tensions were most visible where clinical service expectations and publication expectations were both institutionally salient.

To recruit participants, we used a two-stage procedure. At each institution, a faculty administrator distributed a standardized recruitment notice that described the study purpose, eligibility conditions, confidentiality protections, voluntary participation, and the research team’s contact information. Interested candidates contacted the research team directly. Faculty administrators did not select, screen, rank, or recommend specific trainees, and faculty members were not informed which candidates ultimately participated. This separation between notice distribution and participant enrollment was used to reduce hierarchical pressure and the possibility that faculty contacts would nominate only highly visible or compliant students. Eligibility criteria included: (1) current enrollment as a PhD candidate in a clinical medicine-related field, (2) possession of an initial medical degree (for example, MBBS or an equivalent qualification) and prior clinical training experience, and (3) willingness to discuss educational and career experiences. Candidates in PhD programs without a clinical training component, candidates who had withdrawn from clinical practice for more than two consecutive years before enrollment, and candidates unable to complete the interview in Mandarin Chinese were excluded. No candidate was excluded on the basis of gender, age, or clinical specialty. Maximum variation was operationalized by tracking gender, doctoral year, clinical specialty, and research output after each enrollment [35, 36]. We did not use a fixed quota matrix. Instead, the team updated a recruitment log after each interview and directed subsequent outreach toward specialties, doctoral cohorts, or gender categories that were underrepresented at that stage. When multiple eligible candidates from one institution volunteered, we selected the candidate whose profile contributed the greatest additional variation to the accumulating sample.

Ultimately, 30 PhD candidates, one from each institution in the regional sampling frame, were interviewed. Informed consent was obtained individually by the research team before each interview. Participants were told that declining participation or withdrawing after consent would have no academic or professional consequence. Institutional names are withheld from all publications to protect both participants and local training programs. Participants ranged from first-year to fourth-year doctoral students and represented clinical fields including surgery, internal medicine, pediatrics, obstetrics and gynecology, oncology, neurology, orthopedics, cardiology, and emergency medicine. Table 1 provides an overview of participant characteristics, including affiliated university region, clinical specialty, gender, doctoral year, and research output. Each participant is identified by a code from P01 to P30.

**Table 1:** Characteristics of the 30 interview participants

Participant (Code)	University (Region)	Clinical Field	Gender	Doctoral Year	Research Outputs (Publications)
P01	University E5 (East)	Neurology	Male	Year 4	3
P02	University N15 (North)	Orthopedics	Male	Year 4	3
P03	University N11 (North)	Neurology	Female	Year 1	0
P04	University E12 (East)	Obstetrics/Gynecology	Female	Year 1	0
P05	University E8 (East)	Oncology	Male	Year 3	1
P06	University N7 (North)	Cardiology	Female	Year 2	0
P07	University N6 (North)	Oncology	Male	Year 3	3
P08	University N13 (North)	Pediatrics	Female	Year 2	0
P09	University N12 (North)	Public Health	Female	Year 3	1
P10	University E1 (East)	Cardiology	Female	Year 3	2
P11	University N10 (North)	Pediatrics	Female	Year 4	3
P12	University E11 (East)	Obstetrics/Gynecology	Male	Year 2	0
P13	University E15 (East)	Public Health	Male	Year 1	0
P14	University E7 (East)	Surgery	Female	Year 4	3
P15	University E2 (East)	Emergency Medicine	Male	Year 1	0
P16	University E13 (East)	Surgery	Male	Year 4	2
P17	University N2 (North)	Surgery	Male	Year 4	3
P18	University N14 (North)	Cardiology	Female	Year 3	1
P19	University E4 (East)	Internal Medicine	Female	Year 4	2
P20	University N3 (North)	Internal Medicine	Male	Year 1	0
P21	University E3 (East)	Pathology	Male	Year 2	2
P22	University E14 (East)	Obstetrics/Gynecology	Male	Year 3	2
P23	University N5 (North)	Neurology	Male	Year 2	0
P24	University E10 (East)	Orthopedics	Female	Year 3	2
P25	University N8 (North)	Internal Medicine	Male	Year 4	3
P26	University N9 (North)	Radiology	Female	Year 3	2
P27	University E9 (East)	Surgery	Male	Year 2	0
P28	University N1 (North)	Radiology	Female	Year 2	0
P29	University N4 (North)	Pediatrics	Female	Year 3	1
P30	University E6 (East)	Oncology	Female	Year 2	0

### Reflexivity

The research team comprised scholars in education, public administration, and public affairs with training in qualitative research. No team member held clinical qualifications or had supervisory authority over the participants. The team occupied an insider-outsider position: members were familiar with Chinese higher education institutions and doctoral training systems, but they were outsiders to clinical departments and hospital hierarchies. During coding, each researcher wrote analytic memos that recorded expectations, uncertainties, and cases that challenged provisional interpretations. Biweekly peer debriefings were used to examine whether a code reflected the participant’s account or the coder’s prior assumptions. During type construction, cases that straddled two types were retained as boundary cases and were used to test the sharpness of the type definitions.

### Data collection

Data were collected through semi-structured in-depth interviews. An interview guide was developed to elicit narratives about participants’ educational and professional experiences, focusing on the formation of clinician-scientist identity [37]. Key questions asked participants to describe motivations for pursuing a PhD, weekly allocation of clinical and research work, interactions with mentors and peers, conflicts encountered during training, responses to evaluation requirements, access to institutional support, and envisioned future career paths. Follow-up prompts asked participants to provide concrete episodes and turning points that shaped their professional self-understanding. The interview protocol was reviewed by an expert panel of medical educators for face validity and then pilot tested. Before formal data collection, four pilot interviews were conducted with medical PhD candidates from two institutions outside the final sample. Pilot testing led to two changes: abstract identity questions were replaced with behavioral prompts, and a dedicated mentorship section was added after pilot participants repeatedly discussed mentor relationships. During formal data collection, probing strategies were refined as themes emerged, while the core interview domains remained stable after the fifth interview. The full interview guide is provided in Supplementary Appendix A.

All interviews were conducted one-on-one by the first author, who is trained in qualitative interviewing. For convenience and to encourage openness, most interviews took place via video conferencing (owing to the geographic distribution of participants). Each interview lasted approximately 60 to 90 min. Interviews were conducted in Mandarin Chinese, which was the participants’ native language as well as the interviewer’s. All sessions were audio-recorded with permission and later transcribed verbatim. The Chinese transcripts were then translated into English by a professional bilingual translator, and the translations were cross-checked against the originals by the research team to ensure accuracy and preserve meaning, especially for any culturally specific expressions [38].

### Data analysis

We employed an ideal-typical analysis approach to examine the interview data [39]. First, the research team read all transcripts closely and performed open coding to identify recurring themes and concepts related to professional identity, motivations, role conflicts, supports, and strategies. Using qualitative data analysis software (NVivo 12), codes were systematically applied and refined through constant comparison across interviews [40]. In this initial phase, a coding schema was developed capturing key dimensions such as “primary career motivation,” “self-description of identity,” “perceived conflicts between roles,” “mentor influence,” “views on evaluation metrics,” and “future career intentions.” Two researchers coded the first ten transcripts independently and discussed discrepancies to calibrate the code definitions, ensuring reliability. The remaining transcripts were then coded with this refined scheme, with periodic team meetings to resolve any new code-related questions.

After thematic coding, we shifted to constructing ideal types. Ideal types, in the sociological sense, are analytic constructs that accentuate patterned features of empirical cases to support comparison [41]. In our analysis, each participant’s narrative was treated as a case of clinician-scientist identity formation. The team compared cases across coded dimensions and grouped narratives that shared similar configurations of professional self-definition, motivation, time allocation, mentor relationship, evaluation pressure, and future career intention. We first considered several alternative typologies, including a two-type distinction between integrated and non-integrated identities, a three-type distinction based on dominant career orientation, and a matrix organized by high or low clinical engagement and high or low research engagement. These alternatives were rejected because the two-type scheme concealed differences between clinician-first and research-first orientations, the three-type scheme absorbed persistent ambivalence into residual categories, and the engagement matrix described behavior without adequately capturing identity work. The four-type solution was retained because it best accounted for both dominant orientation and unresolved tension while preserving the comparative boundaries observed across the interviews.

Ultimately, we identified four ideal types of clinician-scientist identity development among the PhD trainees. For each ideal type, we summarized its defining characteristics (e.g., predominant professional orientation, underlying motivations, typical challenges, and patterns of engagement with clinical and research work). We also noted how many participants roughly exemplified each type. These ideal types are reported in the Findings, illustrated with verbatim quotations from participants (with identifying codes) to provide evidence for each type. Pseudonyms were not used; instead, we refer to participants by their code (P01, P02, etc.) alongside basic contextual information when needed (such as their year of study or field) to give the reader relevant background without compromising anonymity [42].

We concluded that the typology was sufficiently refined through iterative team consensus and negative-case review. Each type rested on multiple cases. Adjacent types could be distinguished along motivation, time allocation, mentor relationship, self-definition, and future intention without relying on a single dimension. When the team returned to the full dataset, all cases could be placed within the four-type structure. Analytic sufficiency was therefore defined as the point at which additional comparison clarified boundary conditions without requiring a fifth type or a merger of existing types.

### Research ethics

This study was approved by the Institutional Review Board of Zhejiang University. All participants provided informed consent before taking part in the interviews. They were assured that their responses would be kept confidential and that any publications would not reveal personal identities or specific institutional affiliations. The use of numeric codes (P01-P30) in reporting ensures participant anonymity. Interview recordings and transcripts were stored securely, accessible only to the research team. In presenting the results, we have altered or omitted any potentially identifying details from quotations while striving to maintain the integrity of participants’ accounts.

### Findings

Through our analysis, we identified four ideal-typical pathways of clinician-scientist identity development among the PhD candidates. These ideal types capture distinct combinations of how participants viewed their professional role, why they pursued dual training, and how they navigated the demands of clinical practice and research. Table 2 summarizes the key characteristics of each ideal type, which we have labeled as: (1) Integrated Dual-Identity, (2) Clinician-First (Practice-Focused), (3) Research-First (Science-Focused), and (4) Ambivalent (Struggling to Integrate). Below, we describe each ideal type in detail and provide illustrative quotations from participants (using their participant codes) that exemplify their experiences.

**Table 2:** Ideal types of clinician-scientist identity development among medical PhD candidates

Ideal Type	Identity Orientation	Primary Motivation	Key Challenges/Features
Integrated Dual-Identity (*n* ≈ 8)	Embraces dual identity; sees research and patient care as synergistic	Intrinsic passion for both science and medicine; desire to innovate in clinical care	Manages heavy dual workload by integrating research with practice; leverages cross-role synergies
Clinician-First (Practice-Focused) (*n* ≈ 7)	Clinician identity dominant; research is secondary	Driven by clinical career goals or credential needs; values research mainly to improve practice	Lower research engagement; meets minimum publication requirements; prioritizes clinical duties over lab work
Research-First (Science-Focused) (*n* ≈ 7)	Scientist identity dominant; clinical role is secondary	Passion for discovery and academic career; aims for future in research	Limited clinical engagement; devotes majority of time to lab; risk of losing clinical skills
Ambivalent (Struggling to Integrate) (*n* ≈ 8)	Uncertain or conflicted identity between clinician and scientist	Mixed or unclear motivations; often external pressure or evolving goals	Severe role conflict and stress; difficulty committing to either path; risk of burnout or exit

### Type 1: integrated dual-identity (“Bridge Builders”)

The first ideal type, which about eight of the thirty participants reflected, is the Integrated Dual-Identity pathway. These individuals earnestly embraced both clinician and scientist roles, viewing the two roles as mutually reinforcing. Participants of this type often described themselves as bridge builders between the clinic and the lab. They typically entered the PhD program with a genuine passion for research and a strong commitment to patient care. Compared with participants whose narratives emphasized a single professional domain, Integrated Dual-Identity participants sought ways to make clinical work inform research and to make research improve clinical judgment. Many in this group cited early positive role models, such as a supervisor or senior doctor who was a successful clinician-scientist, or formative experiences that sparked dual interests.

Integrated-type participants were intrinsically motivated by the potential synergy of combining medicine and science. They commonly expressed that doing both made them better in each domain. One fourth-year oncology PhD student explained that treating patients provided inspiration for his research, saying, “I encounter so many unanswered questions in the ward; those questions drive me to investigate in the lab.” (P05). Another participant likewise highlighted the complementary nature of the two roles:

“Working in both worlds gives me a unique perspective. My time with patients sparks ideas for research, and my findings in the lab could improve how I treat patients. I truly feel I’m training to be both a good doctor and a good scientist at the same time.” (P14).

This integrated mindset helped these participants manage what objectively was a heavy workload. They tended to carefully allocate time to each role, often with institutional support or deliberate scheduling. For instance, some reported that their supervisors allowed or even encouraged a balanced schedule: certain days reserved for clinic duties and others for laboratory work. One third-year neurology student noted that while it was “extremely busy,” he felt supported by his mentor’s understanding of both roles. As a result, participants in the Integrated Dual-Identity group were generally high-performing in their programs: they had solid research outputs (often multiple publications by their final year) while also progressing in clinical training. Importantly, they expressed a clear vision of continuing in dual roles after graduation. Several mentioned aspiring to academic clinician-scientist careers, aiming to become faculty in university hospitals where they could treat patients part-time and run research projects. In summary, the Integrated Dual-Identity ideal type is characterized by a strong dual commitment and a belief that the clinician and scientist in oneself can thrive together.

### Type 2: clinician-first (Practice-Focused)

The second ideal type is the Clinician-First pathway, observed in roughly seven participants. These individuals’ professional identities were anchored predominantly in clinical practice. They viewed themselves first and foremost as future physicians, including specialists or surgeons, with research training taking a secondary and supportive role. Compared with the integrated type, Clinician-First participants often viewed the PhD as a credential for advancing a medical career or meeting requirements for employment at a top-tier hospital. Their primary motivation for enrolling in a doctoral program centered on pragmatic career considerations and the belief that research experience could strengthen clinical practice.

Clinician-First participants frequently spoke about how their heart lay in patient care. A third-year surgical trainee (P27) exemplified this sentiment: “I’m a surgeon at heart. Research is useful, but if I have to choose, I’d rather be in the operating theatre than at a lab bench.” (P27). Another participant (P20), a first-year internal medicine PhD student, candidly noted, “To be honest, I enrolled in the PhD because nowadays you almost need one to get a good hospital position. I do find some of the research interesting, but my priority is becoming an excellent clinician.” (P20). Such comments reflect a prevailing view in this type that clinical excellence was the ultimate goal, and research was an adjunct or a stepping stone.

Because of their practice-focused orientation, many Clinician-First students balanced their time heavily in favor of clinical activities. Some continued to take hospital shifts or engage in residency training alongside their PhD research, which often led to time conflicts. One participant (P08) described frequently rushing from ward duties to lab meetings and feeling that “the lab work sometimes takes a backseat when I have patients to see.” These individuals tended to meet the minimum requirements of their research programs (for instance, ensuring they had a publication or two for graduation), but they were less involved in extra research initiatives beyond what was necessary. Several admitted that they struggled to stay enthusiastic about their research projects, especially when clinical responsibilities loomed large. Nonetheless, they recognized the value of understanding research and appreciated skills like reading literature critically, which they believed would inform their clinical practice.

In terms of outcomes, Clinician-First type participants often planned to return to full-time clinical roles after completing the PhD, with limited interest in research-intensive academic careers. They sometimes expressed guilt or worry about dedicating less energy to research than some peers. However, they generally felt justified in their focus: as one fourth-year participant (P02) put it, “At the end of the day, my patients come first. The PhD is important, but it’s one part of my training. My real identity is as a doctor.” (P02).

### Type 3: research-first (Science-Focused)

The third ideal type identified is the Research-First pathway, which characterized about seven of the interviewees. Participants in this category had an identity orientation largely centered on being scientists or researchers, with their physician role in the background. Many of them developed a strong passion for research either during medical school or through early research experiences, which eventually led them to pursue a PhD and consider a career in academia or industry research. These individuals often spoke about the excitement of discovery and the intellectual satisfaction of scientific work, in contrast to what they saw as the more routine or constrained nature of clinical practice.

Research-First participants were typically proactive in research activities and ambitious in their goals. For example, one participant (P07), a third-year oncology PhD candidate, described how he voluntarily extended lab hours and took on additional experiments beyond the dissertation project because he was “hooked” by research. “I find working in the lab far more stimulating than the ward,” he said. “In the lab, I feel I’m contributing to knowledge that could help thousands of patients, not just one at a time.” (P07). This view reflected a common belief in the Research-First group: research offered broad, long-term impact that felt more significant than the immediate impact of individual patient care.

In many cases, Research-First students minimized their clinical involvement during the PhD period to concentrate on research. Some reported that their clinical skills took a backseat or became rusty while they focused on experiments and publications. For instance, a fourth-year participant (P17) admitted, “I haven’t been in the clinic for months because I’ve been trying to finish up two papers. Sometimes I worry I’ll forget my clinical knowledge, but right now my priority is to establish myself as a researcher.” (P17). These participants often benefited from mentors whose primary expertise was research, including supervisors with PhD backgrounds. They thrived in well-resourced laboratory environments that enabled them to produce results. Several in this group had impressive research outputs, such as multiple publications or conference presentations, and they took pride in these achievements.

Looking ahead, most Research-First type interviewees intended to continue heavily in research after graduation. Some planned to pursue postdoctoral fellowships or seek faculty positions focusing on research. Others mentioned industry careers (for example, in biomedical companies or drug development) where their medical background would be valuable but the day-to-day work would be research-oriented. While they did not outright dismiss clinical practice - in fact, a few mentioned they might maintain a minor clinical role to “stay in touch” - their passion clearly lay in scientific inquiry. A potential challenge they acknowledged was the need to eventually re-integrate their dual training; a couple of participants noted they would have to “catch up” on clinical practice or re-enter residency after the PhD, which they anticipated could be difficult. Nonetheless, at this stage, their identity and confidence were strongly tied to being researchers making scientific contributions.

### Type 4: ambivalent (Struggling to Integrate)

The fourth ideal type, evident in roughly eight participants, is an Ambivalent pathway marked by struggle and uncertainty in identity development. These PhD candidates found themselves pulled between clinician and scientist roles without a stable sense of integration or a clear preference for one role. They were often unsure whether they truly wanted a clinician-scientist career or whether they had entered the PhD program because of external expectations, institutional norms, or limited alternatives. This ambivalence was evident in narratives of stress, self-doubt, and fluctuating career plans.

Ambivalent participants usually started their doctoral training with the intention of combining clinical and research work, but over time they became uncertain or dissatisfied with how it was unfolding. For some, the reality of juggling ward duties, course requirements, and lab experiments proved much harder than anticipated. One second-year participant (P23) shared, “I thought I could handle doing both, but it’s been a rude awakening. I’m constantly torn; when I’m in the lab I’m worried about my patients on the ward, and when I’m in the clinic I’m anxious about falling behind in research.” (P23). This sentiment of being “neither here nor there” was common in this group. They often felt they were not performing to their own or others’ expectations, which was discouraging.

A major theme for Ambivalent individuals was conflict and lack of support. Several described situations of competing demands; for example, being expected to cover hospital shifts or take overnight call while also under pressure to produce research results. One participant (P19) gave a vivid account:

“Last month I had a week where I was on 24-hour call every other day, and at the same time my lab experiment was at a critical phase. I basically didn’t sleep. My clinical supervisor needed me, my research supervisor needed me; I felt like I was letting both down.” (P19).

This level of strain often led to thoughts of dropping one of the roles. Some Ambivalent type students contemplated pausing or even quitting the PhD, whereas others considered whether they should forgo clinical practice after completion and focus on a simpler single-path career. Notably, their feelings about identity were fluid; a few mentioned that their outlook shifted back and forth. For instance, a third-year student (P09) said that when a research project went well, she became excited about being a scientist, but after a bad week in the lab she would swing back to wanting to just be a clinician, and vice versa.

Mentorship also shaped these cases. Ambivalent participants often lacked mentors who modeled balanced clinician-scientist roles. Some participants received conflicting expectations from different supervisors, and others reported limited guidance for coordinating clinical training with research deadlines. By the final year of their programs, some participants in the Ambivalent group still had not firmly decided on a career focus and hoped that a successful research result or a positive clinical rotation would clarify their direction.

In summary, the Ambivalent ideal type is defined by internal conflict and unresolved identity development. These trainees struggled to identify a sustainable position within the dual training pathway and questioned whether a clinician-scientist career remained viable. Their experiences highlight the personal toll of dual training when institutional support and role expectations remain insufficiently coordinated. The other three types had more defined orientations, including integrated, clinical, or research orientations; the Ambivalent group remained in flux and was more exposed to burnout and reactive career decisions.

These four types are idealized archetypes. Participants approximated a given type to varying degrees, and hybrid features were present in several narratives. Two clinician-first participants described research with the intrinsic enthusiasm more characteristic of the integrated type in selected interview segments. One research-first participant expressed intermittent doubt that resembled the ambivalent pattern. Classification in such cases followed a two-step procedure: two team members independently assigned each participant to a provisional type, and the full team then compared assignments. When assignments diverged, the team returned to the complete transcript and assessed which identity orientation organized the participant’s narrative across motivation, time allocation, mentor relationship, and anticipated career. Final classification was based on the dominant narrative logic, not on a single isolated passage. Cases with mixed features remained in the dataset and served as reference points for refining type boundaries.

## Discussion

Our analysis shows that clinician-scientist identity formation among medical PhD candidates follows multiple ideal-typical pathways shaped by personal aspirations and structural conditions. Some trainees in our study integrated clinical and research roles, becoming the bridge builders that clinician-scientist training often seeks to cultivate [1, 17]. Other trainees leaned toward a clinician-first or research-first orientation, while a substantial subgroup remained ambivalent and vulnerable to disengagement. These findings resonate with broader observations in medical education: doctors with research training do not necessarily identify as clinician-scientists in the same way, and their trajectories can diverge widely [43, 44].

A key contribution of this study is highlighting how structural factors within the training environment influence these identity pathways. The Chinese context of this research is characterized by intense academic expectations, evolving medical education models, and varying degrees of support for dual-role development [21, 33, 45]. However, the mechanisms we identified are relevant globally in clinician-scientist training. In particular, four structural or systemic influences emerged as pivotal in shaping different identity trajectories: evaluation policy, supervisor support, dual-role conflict, and resource access. Table 3 summarizes how these factors tended to manifest for each of the ideal types identified.

**Table 3:** Influence of structural factors on identity development across ideal types

Factor	Integrated Type	Clinician-First Type	Research-First Type	Ambivalent Type
Evaluation Policy	High publication expectations met with support and intrinsic drive	Publication requirements viewed as external obligation; meets minimum criteria	High standards align with ambition; tends to exceed requirements	Pressure to publish amplifies stress; often struggles to meet requirements
Supervisor Support	Encourages both roles; offers balanced mentorship	Emphasizes clinical competence; minimal research pressure from mentor	Focus on research output; clinical training de-emphasized by mentor	Inconsistent or conflicting guidance; insufficient dual-role support
Dual-Role Conflict	Manages schedule proactively; partly protected time for each role	Clinical duties override research time; frequent scheduling conflicts	Minimizes clinical load to focus on research; occasional disconnect from patients	Constantly torn between lab and clinic; high stress from competing demands
Resource Access	Ample resources in lab and clinic; leverages hospital research support	Focuses on clinical resources; underutilizes lab support	Strong lab funding and facilities; limited use of clinical resources	Resource gaps in one or both domains; lack of support aggravates struggle

Evaluation policies and metrics had uneven effects across the four pathways. Top-tier programs imposed strict requirements; for instance, many participants reported pressure to publish in high-impact journals or meet research milestones for graduation and employment [25]. These policies motivated participants with strong research orientations, especially the Integrated and Research-First types, because publication and project requirements aligned with their preferred professional direction [20]. For Clinician-First and Ambivalent participants, evaluation pressure often operated as an external burden [46]. Several clinically oriented participants described publication requirements as a bureaucratic requirement and reported doing the minimum research needed to satisfy program rules. For Ambivalent participants, heavy emphasis on measurable output intensified feelings of inadequacy and burnout [47]. These dynamics suggest that rigid evaluation systems can inspire some trainees while alienating others [48]. A more flexible approach to evaluation may be warranted, including differentiated tracks and recognition of clinical innovation, collaborative research, and team-based contributions in addition to publications [29].

Supervisor and mentorship practices also played a critical role in shaping identity trajectories. Participants who had mentors embodying the clinician-scientist ideal, such as advisors who balanced patient care with research, were more likely to develop an integrated identity because these mentors provided guidance and visible role models [7]. Many Research-First trainees had supervisors whose work centered on laboratory productivity and scientific output. These mentors reinforced a scientist-leaning identity by encouraging sustained research engagement [19]. Some Clinician-First trainees had clinically eminent mentors for whom research occupied a secondary place, and these mentors validated continued prioritization of clinical training. Ambivalent participants described a lack of coordinated mentorship. Some felt caught between a clinical mentor demanding ward presence and a research supervisor demanding laboratory results, with little communication between the two supervisors about the trainee’s total workload. Mentorship that recognizes the dual nature of training, including formal co-mentorship, appears especially important [49]. When supervisors adjusted expectations and coordinated timelines, trainees were better able to sustain both roles. When guidance was one-sided or poorly coordinated, trainees tended to follow the domain emphasized by the mentor or remained unsupported in the Ambivalent pathway.

The inherent dual-role conflict (the competition between time/energy spent on clinical duties versus research work) was another structural challenge that manifested differently across types. In well-supported cases (often the Integrated type), institutions or departments had arrangements to mitigate conflict, such as formal scheduling of protected research time or the expectation that PhD candidates would take a break from heavy clinical rotations [2]. These measures enabled integrated trainees to manage both sets of responsibilities more smoothly. Research-First trainees often informally resolved the conflict by pushing clinical work to the margins during their PhD years; some even arranged to defer certain clinical requirements until after completing their research, thereby reducing immediate conflict (though potentially at the cost of later reintegration challenges). Clinician-First trainees typically resolved conflict in the opposite direction: by scaling back research involvement to concentrate on their clinical training, as reflected in their more limited research hours and outputs. Meanwhile, the Ambivalent group suffered the most from dual-role conflicts because there were few structural accommodations for them. They described being simultaneously expected to perform as full-time researchers and full-time clinicians. This highlights a need for better coordination in training programs. Without official policies addressing the dual-role challenge, it fell on the students to constantly negotiate with supervisors or make personal sacrifices. Our findings underscore that explicit programmatic solutions (like integrated curriculum planning or alternating blocks of clinical and research focus) are vital to prevent overload and identity strain.

Finally, access to resources and support systems emerged as an influencing factor. Resources included financial support, research infrastructure, administrative assistance, and peer communities. Integrated and Research-First participants often participated in well-funded projects or research groups with laboratory infrastructure, technical assistance, and conference support. Such resources enabled research productivity and signaled that the institution valued the research role. Some Integrated participants also described hospital departments that reduced clinical workload during intensive research periods, indicating that administrative resource allocation supported dual-role development. Clinician-First trainees often used clinical resources while engaging less with laboratory networks or conference opportunities, which limited their participation in research communities. Ambivalent participants emphasized the absence of tailored support, including dedicated funding, structured peer groups, and administrative coordination for trainees carrying dual demands. Resources therefore facilitated identity development when institutional arrangements matched dual-role training demands, and resource gaps intensified strain when trainees had to negotiate clinical and research obligations individually [3].

### Implications and recommendations

Addressing the diverse needs of clinician-scientist trainees requires changes at both the programmatic and policy level. Based on our findings, we propose several recommendations to better support medical PhD candidates on dual training pathways:

Reform Evaluation Criteria: Training programs should adopt flexible evaluation metrics that align research output with clinical competence. Programs could replace uniform publication quotas with individualized academic plans that specify expected research products, clinical competencies, and translational contributions. Recognition of collaborative research, clinical innovation derived from research, and team-based scholarly work may keep clinically oriented trainees engaged without lowering academic standards.

Implement Dual Mentorship Models: Each clinician-scientist trainee would benefit from having both a research mentor and a clinical mentor who communicate with each other and jointly oversee the trainee’s progress. This dual-mentor system can ensure that the trainee’s development in both domains is supported and that expectations are aligned. Mentors should be trained to be aware of the dual demands on these students and to provide coordinated guidance.

Provide Structured Time for Dual Training: Institutions should consider formal scheduling solutions, such as protected research time for clinicians in training or designated clinical rotation-free periods during the PhD. Conversely, for those coming from a research focus, structured clinical refreshers or rotations can be scheduled. This prevents the scenario of a trainee being overburdened by concurrent full-time duties. National bodies and universities might also explore integrated curriculum models (such as the “4 + 4” or other combined frameworks) that interweave clinical and research training more coherently.

Increase Financial and Logistical Support: Dedicated funding and resources for clinician-scientist trainees can ease the practical burdens. Stipends or grants that are specifically aimed at dual-role trainees (to support extra years of training or research expenses) would reduce financial disincentives. Additionally, providing access to support services (for instance, administrative help, mental health services, or peer support groups focused on clinician-scientist trainees) can alleviate stress and foster a sense of community and belonging.

Cultivate a Dual-Role Culture: Academic medical centers and hospitals should make the clinician-scientist career path visible in routine training environments. Practical steps include inviting clinician-scientists into clinical case conferences, discussing research questions generated from patient care, and providing forums where trainees can observe how clinical problems are converted into researchable questions. These arrangements help trainees see dual-role work as an attainable professional pathway.

## Conclusion

In conclusion, our study shows that clinician-scientist identity formation among medical PhD candidates is shaped by the interaction between individual orientation and institutional arrangements. In China’s Double First-Class settings, doctoral trainees encounter strong expectations for both clinical service and research productivity. The four ideal types clarify how trainees respond to these pressures through integrated, clinician-first, research-first, or ambivalent pathways. Training reforms should therefore address evaluation rules, mentorship coordination, protected time, and targeted support for trainees experiencing persistent role conflict. Such reforms may strengthen clinician-scientist development in China and inform comparable dual-role training environments internationally.

## Supplementary Information

Supplementary Material 1. Appendix A: interview guide.

## Data Availability

The data that support the findings of this study are available from the corresponding author upon reasonable request.
