# Supplementary material for: Pathways to clinician-scientist identity among medical PhD candidates: a multi-case ideal-typical analysis
Source: BMC Med Educ. 2026 Aug 10;26:1832. doi: 10.1186/s12909-026-09889-y (PMC13753222; doi:10.1186/s12909-026-09889-y)
Supplement: Supplementary file 1 [file 12909_2026_9889_MOESM1_ESM.docx]

## **Supplementary Appendix A: Interview Guide**

The semi-structured interview guide comprised eight thematic domains. Questions served as entry points; probing followed participants’ responses to elicit concrete examples and narrative depth. After pilot testing, the opening domain was revised from abstract identity questions to behavioral prompts, and a dedicated mentorship domain was added.

1. A Typical Week

- Could you walk me through a typical week in your doctoral program?
- How much time do you spend on clinical work, and how much on research, in a typical week?
- What does a typical day look like when you are in the clinic? What about when you are in the laboratory?

2. Motivations for Pursuing the PhD

- What led you to enroll in a doctoral program?
- At the time you applied, how did you envision the role of the PhD in your career?
- Did a mentor, supervisor, or family member influence your decision? In what way?

3. Balancing Clinical and Research Roles

- How do you manage the demands of clinical training and research simultaneously?
- Can you describe a specific situation where clinical duties and research obligations came into conflict? What happened? How did you handle it?
- Do you feel one role takes priority over the other in your daily life? Which one, and why?

4. Perceptions of Evaluation and Institutional Expectations

- What does your institution expect from you in terms of clinical performance? In terms of research output?
- How do these expectations affect the way you allocate your time?
- Do you feel the evaluation criteria in your program are fair to someone trying to do both clinical work and research?

5. Mentorship

- Can you describe your relationship with your primary mentor or advisor?
- Does your mentor have a clinical background, a research background, or both?
- How does your mentor view the balance between clinical training and research?
- Have you received guidance on how to navigate the dual role? From whom?

6. Institutional Support and Resources

- What resources does your institution provide to support your research?
- What resources does your institution provide to support your clinical training?
- Are there any resources you feel are missing that would help you manage both roles?

7. Self-Definition and Future Career Intentions

- When someone asks what you do, how do you describe yourself professionally?
- Five years from now, what kind of work do you see yourself doing?
- Do you see yourself primarily as a clinician, a researcher, or both? Has this changed since you began the PhD?

8. Reflection: Critical Incidents and Turning Points

- Can you recall a specific event or experience during your doctoral training that changed how you think about your career?
- Was there a moment when you seriously questioned whether the dual path was right for you?
- Is there anything else about your experience as a medical PhD candidate that you think is important for us to understand?

Note: The guide was used flexibly. Question order and phrasing were adapted to the flow of each interview. Domains 1 and 5 were revised based on pilot testing feedback; the remaining domains remained stable throughout data collection.
